# Contact Lens Health Week — August 24–28, 2015

**Published:** 2015-08-21

August 24–28, 2015, marks the second annual Contact Lens Health Week. In collaboration with partners from clinical, public health, industry, and regulatory sectors, CDC is promoting healthy contact lens wear and care practices to reduce the risk for eye infections and complications associated with poor contact lens hygiene.

Research following outbreaks of rare but serious eye infections in the United States showed that these types of infections occur most often in contact lens wearers who do not take proper care of their contact lenses and cases. This finding signaled that action needed to be taken to promote safer contact lens wear and care.

A report in this issue of *MMWR* provides an updated population-based estimate of the number of contact lens wearers in the United States. The report finds that there are 40.9 million contact lens wearers aged ≥18 years. It also includes results of a survey that found more than 99% of contact lens wearers report at least one contact lens hygiene habit that could put them at risk for an eye infection, with the majority of respondents reporting behaviors that can raise the risk for eye infection. Nearly one third of contact lens wearers reported ever experiencing a contact lens-related red or painful eye that required a doctor’s visit.

Contact lens wearers represent a significant proportion of the U.S. population, and their contact lens hygiene habits put them at risk for painful, costly eye infections that could lead to vision problems. This year’s observance targets teenage contact lens wearers, who have been associated with lower contact lens compliance and higher risk for serious eye infections. Proper contact lens hygiene habits, supplies, and regular visits to the eye doctor are all essential to keeping contact lens wearers’ eyes healthy. Additional information on Contact Lens Health Week and the proper wear and care of contact lenses is available at http://www.cdc.gov/contactlenses.

